# Emerging Roles of Liver Sinusoidal Endothelial Cells in Nonalcoholic Steatohepatitis

**DOI:** 10.3390/biology9110395

**Published:** 2020-11-12

**Authors:** Kunimaro Furuta, Qianqian Guo, Petra Hirsova, Samar H. Ibrahim

**Affiliations:** 1 Division of Gastroenterology and Hepatology, Mayo Clinic, Rochester, MN 55905, USA; furuta.kunimaro@mayo.edu (K.F.); guo.qianqian@mayo.edu (Q.G.); hirsova.petra@mayo.edu (P.H.); 2 Division of Pediatric Gastroenterology and Hepatology, Mayo Clinic, Rochester, MN 55905, USA

**Keywords:** adhesion, angiogenesis, extracellular vesicles, fibrosis, liver sinusoidal endothelial cells (LSEC), inflammation, NASH, steatosis

## Abstract

**Simple Summary:**

Nonalcoholic fatty liver disease (NAFLD) is a hepatic manifestation of the metabolic syndrome. With the prevalence of obesity and type 2 diabetes, NAFLD is becoming the most common liver disorder worldwide. More than 10% of NAFLD patients progress to an inflammatory and fibrotic form called nonalcoholic steatohepatitis (NASH), which can lead to end-stage liver disease. Liver sinusoidal endothelial cells (LSEC) are highly specialized cells located at the interface between the flowing blood in the liver and the other liver cells. The current review highlights the recent knowledge of the role of LSEC in the development of NASH, and how LSEC change their structure and function during NAFLD progression. Moreover, the review discusses the pathogenic role of nanometer-sized particles called extracellular vesicles that mediate intercellular communication in the NASH liver. The current manuscript has a special emphasis on the role of adhesion molecules expressed on the LSEC surface in the recruitment of circulating leukocytes to the liver, a critical step in liver inflammation in NASH. Furthermore, the review shed some lights on LSEC-targeted potential therapeutic strategies in NASH.

**Abstract:**

Nonalcoholic steatohepatitis (NASH) has become a growing public health problem worldwide, yet its pathophysiology remains unclear. Liver sinusoidal endothelial cells (LSEC) have unique morphology and function, and play a critical role in liver homeostasis. Emerging literature implicates LSEC in many pathological processes in the liver, including metabolic dysregulation, inflammation, angiogenesis, and carcinogenesis. In this review, we highlight the current knowledge of the role of LSEC in each of the progressive phases of NASH pathophysiology (steatosis, inflammation, fibrosis, and the development of hepatocellular carcinoma). We discuss processes that have important roles in NASH progression including the detrimental transformation of LSEC called “capillarization”, production of inflammatory and profibrogenic mediators by LSEC as well as LSEC-mediated angiogenesis. The current review has a special emphasis on LSEC adhesion molecules, and their key role in the inflammatory response in NASH. Moreover, we discuss the pathogenic role of extracellular vesicles and their bioactive cargos in liver intercellular communication, inflammation, and fibrosis. Finally, we highlight LSEC-adhesion molecules and derived bioactive product as potential therapeutic targets for human NASH.

## 1. Introduction

Non-alcoholic fatty liver disease (NAFLD), is a spectrum of diseases that encompass simple steatosis also known as nonalcoholic fatty liver which is thought to be a benign condition as well as the more advanced inflammatory and fibrotic form of the disease known as nonalcoholic steatohepatitis (NASH). NASH has become the most common cause of chronic liver disease worldwide [1]. NASH is a leading cause of end stage liver disease and its complications including hepatocellular carcinoma (HCC). Hence, NASH culminates in a large economic burden and poor health-related quality of life [1,2]. However, to date there is no regulatory agency-approved therapy for NASH and available treatments only aim to control NASH-associated conditions [3]. Thus, mechanism-based therapeutic strategies that reverse established NASH and control the progression of the disease are of the utmost importance. Recently, a consensus of international experts recommended a change in the name for NAFLD to metabolic (dysfunction)-associated fatty liver disease (MAFLD) [4,5]. However, we elected to continue using the term “NAFLD” in the current review for the following two reasons: (1) the current diagnostic criteria of NAFLD were employed in most of the human studies referred in this review; (2) the term “MAFLD” has yet to be widely used in the research community.

Lipotoxicity caused by hepatocellular lipid accumulation, dysfunction, cell death, and deleterious tissue remodeling has been recognized as the main trigger of the inflammatory response in the NASH liver [6,7]. Hepatocytes under lipotoxic stress release extracellular vesicles (EVs), enriched with proinflammatory mediators. These EVs enhance the recruitment of proinflammatory monocytes into the liver and their adhesion to the liver sinusoidal endothelial cells (LSEC) [8,9,10].

**Figure 1:**
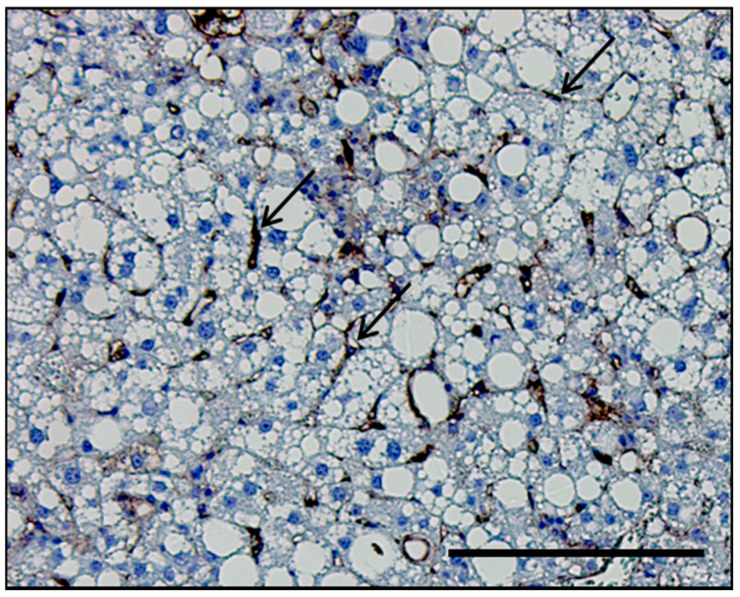
Histological section of the liver of a mouse with nonalcoholic steatohepatitis (NASH). Representative histological section of the liver of a mouse with diet-induced NASH immunostained with the adhesion molecule vascular cell adhesion molecule 1 (VCAM-1), which is predominantly upregulated in LSEC in NASH. VCAM-1 stain delineates LSEC (black arrows) in relation to hepatocytes. Scale bar, 100 μm.

LSEC are highly specialized endothelial cells, arranged as a discontinuous layer to form the vascular bed of the liver sinusoids and separate passenger leukocytes in the sinusoidal lumen from hepatocytes [11]. A histological section of a mouse liver depicting the positional relationship between LSEC and hepatocytes is shown in Figure 1. LSEC uniquely differentiate from other endothelial cells in the body by the lack of basement membrane and the presence of LSEC fenestrae. Fenestrae are 50–200 nm diameter pores covering 2–20% of the endothelial surface. Fenestrae are organized in clusters termed sieve plates that can be altered in response to different stimuli. These features enhance the endocytic capacity of the LSEC and facilitate the elimination of a variety of macromolecules, including lipids and lipoproteins, from the circulation by receptor-mediated endocytosis, making LSEC highly specialized scavenger cells [12]. Moreover, LSEC play a key role in the inflammatory response; they produce chemokines in response to various insults and serve as a platform for various immune cells to lodge in the liver. Therefore, specialized natural killer cells, lymphocytes, and myeloid cells adhere to the surface of LSEC to achieve residence in the liver [11]. LSEC play an anti-inflammatory role early on during NAFLD development by decreasing pro-inflammatory chemokine secretion [13]. On the other hand, as the disease progresses to NASH, impaired LSEC autophagy enhances the expression of chemokines, cytokines, and adhesion molecules, such as C-C motif chemokine ligand 2 (CCL2), CCL5, interleukin-6 (IL-6), and vascular cell adhesion molecule 1 (VCAM-1), and promotes the development of liver inflammation, endothelial-to-mesenchymal transition, and liver fibrosis [14]. In this review, we sought to discuss the emerging role of LSEC in the pathophysiology of NASH by highlighting their protective as well as pathogenic roles with special emphasis on LSEC-adhesion molecules and derived bioactive products as potential therapeutic targets for human NASH.

## 2. LSEC and Liver Steatosis

### 2.1. Physiological Role of LSEC in Lipid Transfer

**Figure 2:**
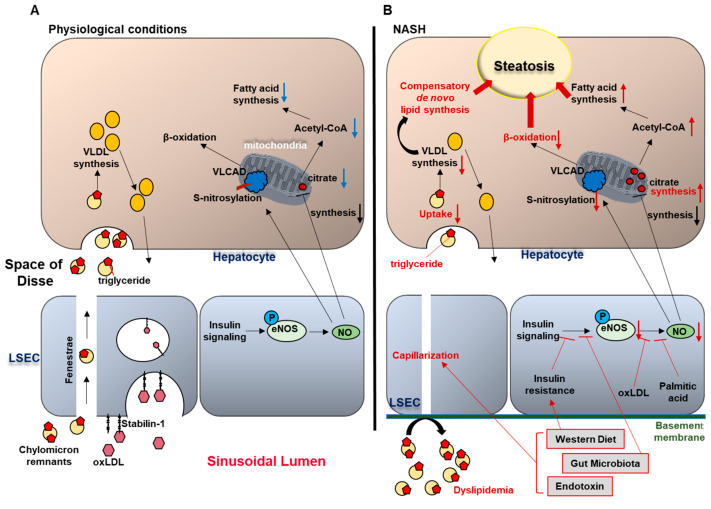
Liver sinusoidal endothelial cells (LSEC) and hepatic steatosis (**A**) under physiological conditions and (**B**) in nonalcoholic steatohepatitis (NASH).

The liver plays a central role in lipid metabolism, including de novo lipogenesis, lipolysis, β-oxidation, and secretion of lipoproteins such as very low density lipoproteins (VLDL) [15]. In physiological conditions, LSEC regulate bidirectional lipid exchange between the blood and the liver parenchyma, and thus play a crucial role in the maintenance of whole-body lipid homeostasis. This task is facilitated by the LSEC open fenestrae without organizing basement membranes, which enable macromolecules and lipoproteins to pass through the endothelial sieve from the sinusoidal lumen to the space of Disse, to be taken up by hepatocytes [16,17]. Hence, chylomicron remnants (triglyceride-depleted but cholesterol- and retinol-rich lipolytic products of chylomicrons), pass through the LSEC fenestrae, and are rapidly taken up by hepatocytes; whereas larger lipoproteins such as triglyceride-rich parent chylomicrons are prone to be trapped in the sinusoidal lumen [18,19,20]. Given that hepatocytes require triglycerides carried by chylomicron remnants for VLDL synthesis; this size-based selective trans-endothelial transport system mediated by LSEC fenestrae plays a crucial role in lipid metabolism under physiological conditions. Moreover, LSEC exhibit a high endocytic capacity, and contribute to the transfer of excess plasma lipids from the circulation to the liver parenchyma [21]. Li et al. reported that mildly oxidized LDL (oxLDL) in plasma is endocytosed by LSEC but not by Kupffer cells. This function of LSEC as a lipid scavenger is mainly mediated by the transmembrane receptor stabilin-1 on the LSEC surface [22] (Figure 2). Since oxLDL is implicated in the pathogenesis of atherosclerosis mainly in patients with diabetes [23,24], LSEC endocytosis may have a protective role against cardiovascular disease and NAFLD progression in diabetes.

### 2.2. LSEC Capillarization and Liver Steatosis

During chronic liver disease, the LSEC lose their fenestrae and form a basement membrane on their abluminal surface. These phenotypic changes in LSEC are referred to as capillarization. Emerging evidence suggests that LSEC capillarization occurs early on during NAFLD pathogenesis [25,26]. Indeed, Miyao et al. observed sinusoidal capillarization in mice as early as one week after the start of choline-deficient, L-amino acid-defined diet using scanning electron microscopy [26]. The authors also detected LSEC capillarization in high fat diet (HFD)-induced NAFLD model without associated severe liver fibrosis. In contrast, Kus et al. recently reported that LSEC fenestrae stayed preserved or even increased their diameter in mice after 20 weeks feeding with HFD, possibly because of compensatory mechanisms to allow excessive plasma fat influx to the liver [27]. Hence, detailed phenotypic characterization of LSEC fenestrae during each phase of NASH progression in animal models that phenocopy the human disease remains to be explored.

Nonetheless, accumulating evidence supports the involvement of nutrient excess diet in sinusoidal capillarization. Zhang et al. showed that oxLDL caused a decrease in the diameter and number of fenestrae in human LSEC in vitro using scanning electron microscopy [28]. Likewise, O’Reilly et al. showed that fasting for 48 h in rats resulted in increased diameter of LSEC fenestrae [29]. Subsequent work by the same group demonstrated that higher fat intake in mice was associated with reduced fenestration frequency and porosity using 25 experimental foods with different dietary components, albeit caution is advised when interpreting this study since relatively old mice (15 months) were employed, given the aging focus [25]. Interestingly, in the same study, the effect of gut microbiota on LSEC fenestration was discussed. Fenestration diameter was positively correlated with the abundance of *Firmicutes* phylum in the cecum, and negatively correlated with that of *Bacteroidetes* phylum. *Bacteroidetes* phylum is known to be increased, while *Firmicutes* phylum is decreased in obese and NASH patients, when compared to healthy controls [30]. This finding is in line with previous report describing endotoxin-induced decrease in the diameter and number of LSEC fenestrae in rats [31]. Moreover, recent evidence has established a profound association of gut-dysbiosis with NASH [32]. Taken together, these data support that both excessive dietary nutrients and gut microbiota-related factors might contribute to LSEC capillarization during NASH pathogenesis.

In addition, accumulating data implicate LSEC capillarization in promoting hepatic steatosis. Indeed, Herrnberger et al. demonstrated that mice lacking liver sinusoidal fenestrations due to genetic deletion of plasmalemma vesicle-associated protein (PLVAP), an endothelial-specific membrane glycoprotein, developed extensive hepatic steatohepatitis along with severe hyperlipoproteinemia under normal chow-fed conditions [20]. In this report, the authors speculated that in mice with lost fenestrae, chylomicron remnants which were required for VLDL synthesis, were trapped in the circulation and could no longer reach the hepatocytes, causing compensatory de novo lipogenesis by hepatocytes, resulting in hepatic steatosis (Figure 2). However, the exact mechanisms of how LSEC capillarization contributes to hepatic steatosis have not been clearly elucidated.

Hence, these reports suggest that excess lipid intake and gut dysbiosis might induce LSEC capillarization, which in turn promotes hepatic steatosis, creating a vicious cycle, although the detailed molecular mediators are an area ripe for further investigation (Figure 2).

In physiological conditions (A), circulating chylomicron remnants are taken up by the liver sinusoidal endothelial cells (LSEC) through the endothelial fenestrae, and delivered to the hepatocytes. In the hepatocytes, triglycerides (TG) carried by chylomicron remnants are used for very low density lipoprotein (VLDL) synthesis. In addition, LSEC endocytosis mediated by the trans-membrane receptor stabilin-1 facilitates the transfer of oxidized LDL from the circulation to the hepatocytes. In NASH (B), western diet and gut microbiota-related factors induce LSEC capillarization. Gut-derived TG-rich chylomicron remnants fail to pass through the endothelial fenestrae and accumulate in the circulation, causing hyperlipidemia. Limited supply of TG to the hepatocytes impairs VLDL synthesis, which induces compensatory de novo lipogenesis, thereby promoting hepatic steatosis. Western diet, insulin resistance, and gut microbiota contribute to the reduced endothelial nitric oxide (NO) bioavailability, and increased intrahepatic vascular resistance. Reduced NO availability enhances hepatic steatosis through: (1) increased fatty acid synthesis (secondary to enhanced mitochondrial citrate synthesis), and (2) impaired β-oxidation (secondary to reduced S-nitrosylation of very long chain acyl-CoA dehydrogenase (VLCAD)).

### 2.3. Endothelial Dysfunction and Liver Steatosis

Fatty liver is known to induce an increase in portal pressure and intrahepatic vascular resistance even in the absence of inflammation and fibrosis, and is considered a high risk for graft failure in donor livers with severe steatosis [33,34,35,36,37]. Indeed, Francque et al. showed increased intrahepatic resistance in diet-induced NAFLD model in rats, using in situ liver perfusion methodology [34]. The increased vascular resistance was partially attributed to reduced size of the sinusoidal space due to the compression by swollen hepatocytes with fat accumulation and ballooning which may impair sinusoidal flow and generate shear stress, disrupting sinusoidal microcirculation [37]. Additionally, increased intrahepatic vascular resistance induces a pathological condition termed endothelial dysfunction. Endothelial dysfunction is defined as the inability of the endothelium to promote vasodilation in response to extrinsic stimuli, primarily as a consequence of impaired production of the endothelium-derived vasodilator nitric oxide (NO) [38]. Accumulating studies using diet-induced fatty liver animal models indicate that hepatic endothelial dysfunction develops during NAFLD and early stages of NASH [39,40,41,42]. A well characterized mechanism that leads to systemic endothelial dysfunction in the metabolic syndrome is insulin resistance, since insulin triggers AKT-dependent endothelial nitric oxide synthase (eNOS) phosphorylation, which mediates endothelial NO synthesis and release [43]. Pasarín et al. examined this mechanism of endothelial dysfunction in NAFLD using their western diet-fed rat model. Moreover, oxLDL or palmitic acid reduced NO bioavailability in vitro [28,44]. Interestingly, a recent study using fecal transplantation in rats showed that restoration of healthy microbiota ameliorated NASH-related portal hypertension, which was associated with the improvement of insulin sensitivity to endothelial NO synthesis signaling, suggesting the possible involvement of gut microbiota in liver endothelial dysfunction in NASH [42]. To the best of our knowledge, there have been no reports showing that increased intrahepatic vascular tone per se influences the development of liver steatosis. Nevertheless, decreased NO bioavailability has been shown to induce hepatic steatosis. Indeed, Schild et al. demonstrated massive hepatic fat accumulation in mice lacking eNOS when fed a normal diet [45]. In this study, eNOS^−/−^ mice also showed a relative excess of mitochondrial citrate synthase activity, which could be a major cause of enhanced hepatic steatosis, since citrate promotes Acetyl-CoA-mediated fatty acid synthesis [46]. In addition, Cohen et al. reported that eNOS-derived NO induced S-nitrosylation of the liver enzyme very long-chain acyl-CoA dehydrogenase (VLCAD), which regulates β-oxidation of fatty acid in mitochondria [47]. Collectively, a vicious cycle between hepatic steatosis and reduced NO bioavailability contributes to the impaired intrahepatic microcirculation in NASH (Figure 2).

## 3. LSEC and Liver Inflammation in NASH

### 3.1. Anti-Inflammatory Roles of LSEC in an Early Stage of NASH

**Figure 3:**
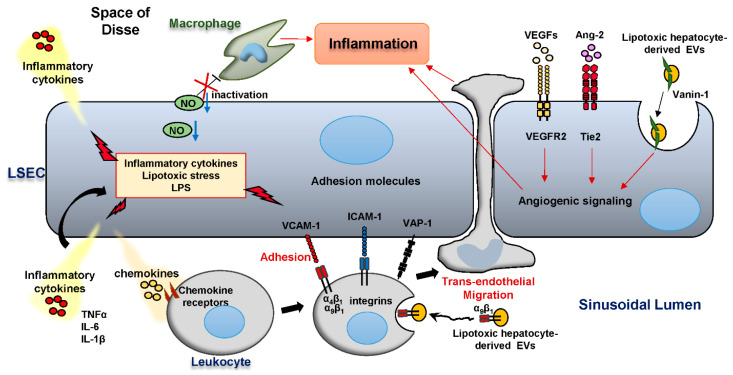
LSEC and hepatic inflammation in NASH.

Along with hepatic steatosis, inflammation is another key hallmark of NASH. LSEC intrinsically exhibit anti-inflammatory functions, preventing excessive activation of the immune system in the liver. Indeed, LSEC exert antigen presenting capacity to naive CD4^+^ T cells, which leads to T cell differentiation towards a regulatory phenotype [48]. Additionally, LSEC efficiently uptake circulating antigens followed by a cross-presentation of these antigens to CD8^+^ T cells. Thus, LSECs antigen presentation results in decreased T cell cytotoxicity, thereby contributing to hepatic immune tolerance [49]. Furthermore, LSEC are responsible for scavenging most of the circulating gut bacteria-derived lipopolysaccharide (LPS), and chronic exposure of LSEC to LPS leads to reduced nuclear factor-κB (NF-κB) signaling, which results in reduced leukocyte adhesion to LSEC, supporting the notion that LSEC prevent hepatic inflammation caused by the gut microbiome-related products [50,51]. Likewise, Tateya et al. showed that mice fed a high fat diet (HFD) for 4 weeks, mimicking the early stage of NASH, had reduced hepatic NO bioavailability, while treatment with the NO signaling enhancer sildenafil in mice fed the HFD for 8 weeks reduced hepatic inflammation [39]. In addition, mice lacking eNOS, which generates endothelial NO, had increased macrophage-associated hepatic inflammation even when fed a low fat diet [39]. These observations indicate that LSEC-derived NO plays an anti-inflammatory role in NASH (Figure 3).

In NASH, reduced nitric oxide (NO) bioavailability drives hepatic inflammation. Proinflammatory cytokines and chemokines, in addition to lipotoxic stress, and microbiome-related products promote LSEC release of inflammatory mediators including tumor necrosis factor alpha (TNFα), interleukin (IL)-6, IL-1β. Integrin α_9_β_1_ is enriched in lipotoxic hepatocyte-derived extracellular vesicles (EVs) and promotes monocyte adhesion to LSEC via its binding interaction with the adhesion molecule vascular cell adhesion molecule-1 (VCAM-1) on the LSEC surface. Intercellular adhesion molecule 1 (ICAM-1) and the monoamine oxidase vascular adhesion protein-1 (VAP-1) also function as adhesion molecules, which mediate leukocyte homing in inflamed liver. The vascular endothelial growth factor (VEGF) and angiopoietin/Tie2 pathways are activated in NASH and enhance angiogenesis. Furthermore, Lipotoxic hepatocyte-derived EVs are taken up by LSEC in a Vanin-1-dependent manner, and elicit a pro-angiogenic signaling, thereby contributing to hepatic inflammation.

### 3.2. Pro-Inflammatory Roles of LSEC in NASH

As NASH progresses to more advanced stages, LSEC acquire a pro-inflammatory phenotype, including: (1) release of pro-inflammatory mediators including cytokines and chemokines; (2) aberrant expressions of adhesion molecules; and (3) acquisition of angiogenetic properties. All these features are important players in the pathogenesis of NASH (Figure 3) [52,53,54,55]. For example, LSEC expressing the pattern recognition receptor (PRR) Toll-like receptor 9 (TLR9) can uptake the bacterial DNA mimic CpG-oligonucleotides by a scavenger receptor-mediated endocytosis, resulting in the activation of the transcription factor NF-κB and secretion of the inflammatory cytokines interleukin (IL)-1β and IL-6 [52]. In addition, LSEC can respond to various types of TLR ligands by producing cytokines including TNF-α, IL-6, or interferon-β. These response characteristics to each TLR ligand are unique to LSEC when compared to Kupffer cells isolated from the same mice [53]. Furthermore, LSEC secrete in response to lipotoxic treatment, among other insults, various chemokines including C-C motif chemokine ligand (CCL) 1, CCL2, CCL25, and several chemotactic (C-X-C motif) ligands, which can drive the recruitment of leukocytes into the liver parenchyma, a critical step in NASH progression (Figure 3) [54,55]. Hence, these observations suggest that not only professional immune cells (leukocytes) but also LSEC may contribute to the progression of NASH through the production and release of pro-inflammatory mediators.

### 3.3. The Role of LSEC Adhesion Molecules in the Hepatic Leukocyte Recruitment

Recruitment of circulating immune cells into injured tissues is a critical step in the initiation and the propagation of inflammation. Immune cell recruitment is a multi-step process consisting of rolling, adhesion, and trans-endothelial migration of leukocytes, all of which are tightly regulated processes facilitated by specific interactions between adhesion molecules expressed on endothelial cells and their counterparts on leukocytes [56]. In the liver, LSEC also play an important role in leukocyte recruitment. Expression profiles of adhesion molecules in LSEC are unique among other types of endothelial cells in that: (1) expressions of selectins, which are mainly responsible for leukocyte rolling, are minimal in vivo, presumably because initial recruitment step does not require rolling due to the narrowness of the sinusoidal lumen [57,58]; and (2) in addition to conventional adhesion molecules including VCAM-1 and intracellular adhesion molecule-1 (ICAM-1), LSEC employ atypical adhesion molecules such as vascular adhesion protein-1 (VAP-1) and stabilin-1 to promote leukocyte recruitment [56]. Characteristics, functions and clinical relevance of major adhesion molecules expressed on LSEC are shown in Table 1. Indeed, growing evidence suggests that these adhesion molecules are overexpressed and play a key role in various pathologic conditions in the liver (Figure 3) [59]. An in vitro study using blocking antibodies to several adhesion molecules showed that lymphocyte adhesion to TNF-α-stimulated primary human LSEC was dependent on ICAM-1, VCAM-1, and VAP-1 [60]. Furthermore, Patten et al. reported that in addition to the conventional paracellular route, circulating lymphocytes can cross directly through the body of LSEC referred to “intracellular crawling”. This observation was made utilizing electronic microscopic examination of patient livers and in vitro live cell imaging in conjunction with flow-based adhesion assays. This mechanism of migration is mediated by the adhesion molecules ICAM-1 and stabilin-1 as demonstrated by an in vitro experiment using neutralizing antibodies to each of these adhesion molecules [61]. Likewise, Weston et al. demonstrated that genetic deletion or pharmacological blockade of VAP-1 resulted in reduced leukocyte recruitment to the liver and attenuated fibrosis in diet-induced NASH mouse model [62]. They also showed that serum soluble VAP-1 levels are elevated in patients with NAFLD compared to healthy controls [62]. Furthermore, we have recently reported using in vitro shear stress adhesion assay system that the adhesion molecule integrin (ITG) α_9_β_1_, which is an abundant cargo of lipotoxic hepatocyte-derived extracellular vesicles (EVs), mediates monocyte adhesion to LSEC via its binding interaction with LSEC VCAM-1 (Figure 3) [10]. This finding is in line with a previous report showing that elevated serum VCAM-1 levels in NAFLD patients significantly correlate with the extent of liver fibrosis [63]. We also demonstrated that neutralizing antibody against the VCAM-1 ligand ITGβ_1_ ameliorated diet-induced NASH in mice [10]. Likewise, Miyachi et al. showed that neutralizing antibody against another VCAM-1 ligand ITGα_4_ inhibited adhesion and migration of monocytes and improved liver inflammation in murine NASH model, further implicating VCAM-1 in the inflammatory process in NASH pathogenesis [55]. Interestingly, they also showed that treatment of mouse LSEC with the lipotoxic free fatty acid palmitate can induce overexpression of adhesion molecules, including ICAM-1 and VCAM-1, suggesting that metabolic stress can directly trigger the upregulation of adhesion molecules in LSEC [55]. Similarly, Rai et al. recently examined the role of ITGα_4_β_7_ in CD4 T cell recruitment to the liver as well as the gut in NASH using junctional adhesion molecule A-deficient mice-fed a western diet [64]. Nevertheless, the LSEC signaling mechanisms responsible for lipotoxic stress-induced expression of adhesion molecules in LSEC have not been elucidated yet, hence further research is needed to fill this knowledge gap. Collectively, LSEC mediate leukocyte homing in the liver by overexpressing various adhesion molecules, thereby promoting liver inflammation in NASH (Table 1).

### 3.4. Angiogenesis Accelerates Liver Inflammation

During chronic inflammation, high levels of angiogenic factors such as vascular endothelial growth factors (VEGFs) and angiopoietins promote blood vessel formation [72]. Moreover, abnormal or excessive vascular growth enhances the inflammatory responses in various disorders including atherosclerosis, inflammatory bowel disease, and rheumatoid arthritis [73]. In the liver, LSEC also play a major role in angiogenesis through VEGF/VEGF receptor (VEGFR) and angiopoietin/tyrosine kinase with immunoglobulin-like and EGF-like domains 2 (Tie2) [74,75]. Indeed, several in vivo studies suggest that the angiogenic signaling accelerates the inflammatory response and fibrosis during liver injury [76,77,78]. Likewise, in the pathogenesis of NASH, emerging evidence implicates aberrant activation of angiogenic signaling in the inflammatory process of the disease (Figure 3). Coulon et al. demonstrated that pharmacological blockade of VEGFR2 reduced hepatic inflammation in MCD-fed mice [79]. This study also showed that anti-VEGFR2 antibody treatment increased the hepatic gene expression of *Scd1*, which has a key role in partitioning excess lipid into monounsaturated fatty acids, leading to reduced hepatic lipid storage in MCD-fed mice [79]. Nonetheless, published studies were contradictory regarding the role of serum level of VEGF in predicting NAFLD severity [80,81]. Furthermore, the level of soluble VEGFR1 but not VEGFR2 was significantly increased in the serum of patients with isolated steatosis and NASH when compared to healthy controls [80]. Thus, further investigations are required to determine the role of serum VEGF or soluble VEGFR levels as potential biomarkers for NAFLD severity. Additionally, caution should be taken in employing anti-VEGF therapies in a clinical setting, since VEGF signaling is essential also in fibrosis resolution and tissue repair in the liver [76]. Meanwhile, Lefere et al. showed that pharmacological inhibition of the angiopoietin-2 (Ang-2)/Tie2 interaction improved vascular morphology and ameliorated steatohepatitis in MCD-induced murine NASH [82]. Interestingly, they also demonstrated that Ang-2 signaling blockade attenuated LPS-induced expression of the adhesion molecule VCAM-1 using endothelial cell line [82]. These findings implicate angiogenic signaling in leukocyte-endothelial cells adhesion, an initiating process in the inflammatory response. Furthermore, serum Ang-2 levels were increased in patients with NASH and correlated with the extent of hepatic steatosis, inflammation and hepatocyte ballooning [82]. Hence, numerous in vivo preclinical and clinical studies support the contribution of angiogenesis to the liver inflammation in NASH.

## 4. The Role of LSEC in NASH-Related Fibrosis

Liver fibrosis is an excessive accumulation of extracellular matrix that occurs in most types of chronic liver diseases, including NASH, and likely represents an attempt to repair and replace damaged liver cells. Hepatic stellate cells (HSC), which are found in the perisinusoidal space between hepatocytes and LSEC, are primarily responsible for the extracellular matrix deposition. In a healthy liver, HSC are in a quiescent state. During liver injury or inflammation, profibrogenic cues make HSC undergo a transformation from a quiescent to a proliferative, migratory, contractile, and fibrogenic phenotype, and start to secrete a variety of extracellular matrix proteins, including collagens, glycoproteins and proteoglycans, instigating liver fibrosis [83]. Given their proximity, LSEC and HSC are topographically associated and regulate each other’s phenotype and function via paracrine signaling. HSC (along with hepatocytes) secrete VEGF which maintains LSEC phenotype. On the other hand, LSEC play a major role in maintaining HSC quiescent phenotype via paracrine signaling [84]. One of the signals maintaining HSC quiescence is the release of NO from LSEC, which is regulated by the activity of endothelial NO synthase. Capillarized LSEC do not maintain HSC in a quiescent state, suggesting that phenotypic changes accompanying loss of fenestrae are permissive for HSC activation, and therefore permissive for fibrosis [85]. The LSEC ability to maintain HSC quiescence is impaired during chronic liver injury as LSEC capillarization is associated with a proinflammatory phenotype. It is also likely that there is a positive feedback loop between LSEC dysfunction and HSC profibrogenic activation. For example, HSC-induced tissue stiffness due to extracellular matrix deposition can mechanically activate LSEC [86]. The importance of LSEC in liver fibrosis has been extensively investigated in a variety of models of liver injury [87], however, relatively less is known about the role of LSEC in NASH-related fibrosis.

It has been reported that LSEC capillarization in mouse NASH liver precedes the onset of fibrosis [88]. This may suggest that LSEC may contribute to the initiation of fibrogenesis in fatty liver. As LSEC dysfunction associated with decreased NO production contributes to liver fibrosis, it would be interesting to know whether restoration of NO would attenuate NASH severity. Although it is well accepted that HSC activation occurs as a result of LSEC inability to maintain their quiescence, it is also plausible that dysfunctional or activated LSEC promote HSC activation directly via the release of profibrogenic signals. Indeed, activated LSEC have been shown to release profibrogenic factors such as laminin and fibronectin [89,90].

Prior studies have also implicated the Hedgehog pathway as important paracrine signaling in NASH and liver fibrogenesis. LSEC are responsive to Hedgehog ligands as well as produce Hedgehog molecules [91], which in turn may exert a profibrogenic effect on HSC. The Hedgehog pathway has also been shown to regulate LSEC capillarization [91]. Interestingly, pharmacological inhibition of the Hedgehog pathway by vismodegib (also known as GDC-0449) attenuated liver inflammation and fibrosis in a mouse model of NASH [92]. The therapeutic effect of vismodegib was primarily ascribed to decreased liver injury, but it is also possible that Hedgehog inhibition had an effect on LSEC dysfunction; however, this parameter was not assessed. Overall, future studies warrant attention to NASH-related angiocrine signaling (i.e., endothelial cell-released paracrine molecules, such as growth factors, trophogens, and chemokines) [84], and its role in fatty liver progression. Initial evidence from experimental models suggests that divergent angiocrine signals coming from LSEC initiate liver regeneration during acute insult but promote fibrogenesis upon chronic liver injury unrelated to NALFD. The pro-fibrotic signaling cascade involves fibroblast growth factor receptor 1 and C-X-C motif chemokine receptor 4 as genetic deletion of these genes in the endothelium prevented fibrosis and restored the pro-regenerative response in acute injury [93]. These models underscore the importance of identifying therapeutic strategies that enhance pro-regenerative responses without provoking fibrosis. However, whether this pathway is also involved in NASH-induced liver injury is an area ripe for further exploration.

### Intercellular Communication of LSEC and other Liver Cells via Extracellular Vesicles in NASH Pathogenesis

Cells in multicellular organisms release membrane-derived nanometer-sized particles called extracellular vesicles (EVs). Based on their biogenesis mechanism and size, EVs are mainly classified into exosomes and microvesicles. Exosomes (50–150 nm in diameter) are derived from endosomal compartments called multivesicular bodies and released form the cells via exocytosis, whereas microvesicles (50–1000 nm) are released via direct budding from the plasma membrane [94]. EVs can transport their specific cargo (proteins, lipids, metabolites, and nucleic acids) between cells, thereby playing an essential role in cell-to-cell communication in both physiological and pathological conditions [95,96,97]. Endothelial cells efficiently uptake biologically active EVs. Indeed, recent study employing in vivo live-tracking approaches on zebrafish embryos showed that endogenous EVs are endocytosed by endothelial cells as well as macrophages in the tail, and the uptake of EVs is essential for the normal development of the vascular plexus [98]. Likewise, in the liver, accumulating evidence suggests that EV-mediated intercellular communication of LSEC and other liver cell types plays a pivotal role in various diseased conditions [96,97]. In a rat bile duct ligation model, HSC and cholangiocytes release Hedgehog ligand-containing EVs, which induce a tissue remodeling phenotype in targeted LSEC [99]. Likewise, Lemoinne et al. showed that profibrotic mesenchymal cells residing in the portal area, also called “portal myofibroblasts” release vascular endothelial growth factor A (VEGFA)-containing EVs, which enhance proangiogenesis in LSEC [100]. Moreover, the role of LSEC as EV donor cells has also been studied. Using CCl_4_-induced chronic liver injury mouse model, LSEC-derived EVs were shown to drive pathological HSC migration via the specific EV cargo sphingosine kinase 1 (SK1) [90]. As this study used EVs derived from healthy LSEC, it is tempting to speculate that EVs derived from dysfunctional LSEC, or NASH-associated LSEC, may exert unique effects on HSC, which is yet to be investigated. In the pathogenesis of NASH, Povero et al. showed that free fatty acid-treated HepG2 cells release EVs enriched with the cell surface enzyme protein Vanin-1 (VNN1), and these EVs are efficiently taken up by human umbilical vascular endothelial cells (HUVEC) in a VNN1-dependent manner [101]. They also demonstrated that mice fed a methionine and choline-deficient diet had increased circulating VNN1-containing EVs which induce endothelial tubular formation and chemotaxis in vitro (Figure 3) [101]. These observations indicate that EVs drive LSEC angiogenesis in NASH. Nevertheless, the role of LSEC as EV donors, as well as communication of LSEC via EVs with other cell types in the liver or other organs and systems such as the cardiovascular system and the adipose tissue is still an unexplored area. Further studies in this field will provide new insights into the pathophysiology of NASH and the metabolic syndrome.

## 5. The Role of LSEC in NASH-Associated HCC

Nonalcoholic fatty liver disease (NAFLD) is becoming a major cause of HCC (59%), with a cumulative incidence of 0.3% over a six-year follow-up in the United States [102,103,104]. Interestingly, HCC in the absence of cirrhosis is an increasingly recognized complication of nonalcoholic fatty liver disease and the associated metabolic syndrome including obesity and type 2 diabetes mellitus [103,105].

Angiogenesis and extensive vascular remodeling are the fundamental events in the progression of fibrosis, cirrhosis, and HCC in NASH and other chronic liver diseases [106]. Moreover, peri-tumoral angiogenesis can predict HCC metastasis and patient prognosis. The liver peri-tumoral endothelial cells (PECs) exhibit higher proliferation in response to proangiogenic factors interlukine-6 (IL-6)/soluble interlukine-6 receptor (sIL-6R) co-treatment compared to tumoral endothelial cells (TECs) in HCC. Furthermore, IL-6 secretion by PECs is induced by peri-tumoral hypoxia, and the increased IL-6 levels contribute to PEC proliferation [107]. In addition, endothelial trans-differentiation, characterized by sprouting angiogenesis and loss of LSEC markers (stabilin-1, stabilin-2, lymphatic vessel endothelial hyaluronan receptor 1 (LYVE-1) and CD32b) is a hallmark of HCC progression in both murine and human studies. Interestingly, the loss of stabilin-2 in the PEC was associated with improved patient survival in HCC by preventing endothelial-tumoral cell adhesive interactions and microvascular invasion [108].

Recently, fatty acid binding protein 4 (FABP4), a cytosolic fatty acid chaperone, has been implicated in liver carcinogenesis related to the metabolic syndrome. FABP4 plays a key role in insulin resistance, type 2 diabetes mellitus, and atherosclerosis [109,110,111]. Moreover, FABP4 has been reported to facilitate energy delivery in the form of fatty acids from adipocytes to surrounding breast and ovary cancer cells [112,113]. Milner and colleagues showed that elevated circulating adipocyte fatty acid-binding protein levels in NAFLD patients, can predict inflammation and fibrosis and may have a pathogenic link to disease progression [114]. Interestingly, de novo expression of LSEC FABP4 in response to exposure to glucose, insulin or hypoxia potentiates the oncogenic effects of hepatoma cell lines (HepG2, SKHep1, and Huh7) through activation of mechanistic target of rapamycin (mTOR) pathway. This cross talk between LSEC and hepatoma cells was mainly mediated by FABP4-enriched microvesicles released from endothelial cells. Furthermore, the FABP4 inhibitor (BMS309403) significantly reduced tumor growth in the HepG2 xenograft mice on high fat diet. Interestingly, FABP4 was overexpressed in human HCC samples from patients with NAFLD, when compared with other chronic liver diseases. Moreover, FABP4 expression was restricted to the peri-tumoral endothelial cells [115]. Taken together, these reports support a role of liver endothelial cells, aberrant angiogenesis, transdifferentiation, and expression of FABP4 during NASH in the development of HCC.

## 6. Conclusions and Reflection

Liver sinusoidal endothelial cells have an essential role in maintaining liver homeostasis in physiological conditions and early stage of NAFLD. LSEC capillarization and dysfunction occur early on in the disease process, at the stage of simple steatosis, and might be the defining factor that stratifies patients with simple steatosis and those who progress to NASH. LSEC play a key role in fundamental aspect of NAFLD progression including angiogenesis, inflammation, fibrosis and HCC. Hence, harnessing the immunomodulatory and homeostatic functions of the LSEC might serve as potential therapeutic strategies in NASH; Exploration of this aspect will be feasible with the development of conditional transgenic and knockout mouse models for key pathogenic molecules discussed above. Furthermore, the liver sinusoidal endotheliopathy characterized by increased adhesion molecules expression in NASH is a key element in disease progression, and can be further targeted by the development of small molecules pharmacological inhibitors for various adhesion molecules with liver selectivity.

## Figures and Tables

**Table 1:** Functional roles and clinical relevance of LSEC adhesion molecules and their counterpart ligands in liver diseases.

Adhesion Molecules	Counter-Part Ligands	Preclinical Animal Studies			Clinical Studies
		Loss-of-Function	Animal Model	Hepatic Histological Readout	Adhesion Molecule as Biomarker
**VCAM-1/MAdCAM-1**	ITGα_4_β_1_ ITGα_4_β_7_ ITGα_9_β_1_	α_4_β_1_ blockade	HFD-fed mouse	Reduced myeloid cell accumulation [55]	Serum VCAM-1 level correlates with liver fibrosis score in NAFLD [63]
		α_4_β_7_ blockade	WD-fed mouse	Reduced CD4^+^ T cell recruitment and fibrosis [64]	
		β_1_ blockade	FFC-fed mouse	Reduced MoMF-associated inflammation and fibrosis [10]	
**ICAM-1**	ITGα_L_β_2_ ITGα_M_β_2_	ICAM-1 blockade	I-R injury in rat	Reduced necrosis [65]	Serum ICAM-1 level predicts histological severity in ALD [66]
			Gal/ET-induced shock in mouse	Reduced necrosis [67]	
		α_M_β_2_ blockade	I-R injury in rat	Reduced necrosis and neutrophil recruitment [68]	
**NCAM**	NCAM N-cadherin proteoglycans	NCAM^−/−^	BDL in mouse	Reduced fibrosis [69]	N.A.
**VAP-1**	Unknown	VAP-1 blockade	ConA treatment in mouse	Reduced Th2 cell recruitment [70]	Serum VAP-1 level: (1) predicts histological severity in NAFLD [62], and (2) is increased in ALD and PBC [71]
		VAP-1 blockade VAP-1^−/−^	Diet-induced NAFLD/NASH models, CCl_4_ model in mice	Reduced inflammatory cell infiltrate and fibrosis [62]	

VCAM-1, vascular cell adhesion molecule 1; MAdCAM-1, mucosal address in cell adhesion molecule-1; MoMF; monocyte-derived macrophage; ICAM-1, intracellular adhesion molecule 1; NCAM, neural cell adhesion molecule; VAP-1, vascular adhesion protein-1; HFD, high-fat diet; WD, western diet; FFC, fat-, fructose- and cholesterol-rich diet; I-R; ischemia-reperfusion; Gal/ET, galactosamine/endotoxin; BDL, bile duct ligation; ConA, concanavalin A; CCl_4_, carbon tetrachloride; ALD, alcoholic liver disease; N.A., not applicable; PBC, primary biliary cholangitis.

## Abbreviations

ALD	alcoholic liver disease
Ang-2	angiopoietin-2
CCL	C-C motif chemokine ligand
ConA	concanavalin A
eNOS	endothelial nitric oxide synthase
EV	extracellular vesicle
FABP4	fatty acid binding protein 4
IL-6	interleukin-6
HCC	hepatocellular carcinoma
HFD	high fat diet
HSC	hepatic stellate cells
HUVEC	human umbilical vascular endothelial cells
ICAM-1	intracellular adhesion molecule-1
LDL	low density lipoproteins
LPS	lipopolysaccharide
LSEC	liver sinusoidal endothelial cells
LYVE-1	lymphatic vessel endothelial hyaluronan receptor 1
mTOR	mechanistic target of rapamycin
NASH	nonalcoholic steatohepatitis
NAFLD	nonalcoholic fatty liver disease
NF-κB	nuclear factor-kappa B
NO	nitric oxide
oxLDL	oxidized LDL
PEC	peri-tumoral endothelial cells
PRR	pattern recognition receptor
PLVAP	plasmalemma vesicle-associated protein
sIL-6R	soluble interlukine-6 receptor
SK1	sphingosine kinase 1
TECs	tumoral endothelial cells
TNFα	tumor necrosis factor alpha
Tie2	angiopoietin/tyrosine kinase with immunoglobulin-like and EGF-like domains 2
TLR	toll-like receptor
VNN1	vanin-1
VAP-1	vascular adhesion protein-1
VCAM-1	vascular cell adhesion molecule 1
VEGF	vascular endothelial growth factor
VEGFR2	vascular endothelial growth factor receptor 2
VLCAD	very long-chain acyl-CoA dehydrogenase
VLDL	very low density lipoproteins

## References

[1] Younossi Z.M. (2019). Non-alcoholic fatty liver disease—A global public health perspective. J. Hepatol..

[2] Younossi Z.M., Ratziu V., Loomba R., Rinella M., Anstee Q.M., Goodman Z., Bedossa P., Geier A., Beckebaum S., Newsome P.N. (2019). Obeticholic acid for the treatment of non-alcoholic steatohepatitis: Interim analysis from a multicentre, randomised, placebo-controlled phase 3 trial. Lancet.

[3] Friedman S.L., Neuschwander-Tetri B.A., Rinella M., Sanyal A.J. (2018). Mechanisms of NAFLD development and therapeutic strategies. Nat. Med..

[4] Eslam M., Sanyal A.J., George J. (2020). MAFLD: A consensus-driven proposed nomenclature for metabolic associated fatty liver disease. Gastroenterology.

[5] Fouad Y., Waked I., Bollipo S., Gomaa A., Ajlouni Y., Attia D. (2020). What’s in a name? Renaming ‘NAFLD’ to ‘MAFLD’. Liver Int..

[6] Neuschwander-Tetri B.A. (2010). Hepatic lipotoxicity and the pathogenesis of nonalcoholic steatohepatitis: The central role of nontriglyceride fatty acid metabolites. Hepatology.

[7] Schuppan D., Surabattula R., Wang X.Y. (2018). Determinants of fibrosis progression and regression in NASH. J. Hepatol..

[8] Ibrahim S.H., Hirsova P., Tomita K., Bronk S.F., Werneburg N.W., Harrison S.A., Goodfellow V.S., Malhi H., Gores G.J. (2016). Mixed lineage kinase 3 mediates release of C-X-C motif ligand 10-bearing chemotactic extracellular vesicles from lipotoxic hepatocytes. Hepatology.

[9] Tomita K., Kabashima A., Freeman B.L., Bronk S.F., Hirsova P., Ibrahim S.H. (2017). Mixed lineage kinase 3 mediates the induction of CXCL10 by a STAT1-dependent mechanism during hepatocyte lipotoxicity. J. Cell Biochem..

[10] Guo Q., Furuta K., Lucien F., Gutierrez Sanchez L.H., Hirsova P., Krishnan A., Kabashima A., Pavelko K.D., Madden B., Alhuwaish H. (2019). Integrin beta1-enriched extracellular vesicles mediate monocyte adhesion and promote liver inflammation in murine NASH. J. Hepatol..

[11] Knolle P.A., Wohlleber D. (2016). Immunological functions of liver sinusoidal endothelial cells. Cell Mol. Immunol..

[12] Sorensen K.K., Simon-Santamaria J., McCuskey R.S., Smedsrod B. (2015). Liver sinusoidal endothelial cells. Compr. Physiol..

[13] McMahan R.H., Porsche C.E., Edwards M.G., Rosen H.R. (2016). Free fatty acids differentially downregulate chemokines in liver sinusoidal endothelial cells: Insights into non-alcoholic fatty liver disease. PLoS ONE.

[14] Hammoutene A., Biquard L., Lasselin J., Kheloufi M., Tanguy M., Vion A.C., Merian J., Colnot N., Loyer X., Tedgui A. (2020). A defect in endothelial autophagy occurs in patients with non-alcoholic steatohepatitis and promotes inflammation and fibrosis. J. Hepatol..

[15] Gluchowski N.L., Becuwe M., Walther T.C., Farese R.V. (2017). Lipid droplets and liver disease: From basic biology to clinical implications. Nat. Rev. Gastroenterol. Hepatol..

[16] Hammoutene A., Rautou P.E. (2019). Role of liver sinusoidal endothelial cells in non-alcoholic fatty liver disease. J. Hepatol..

[17] DeLeve L.D., Maretti-Mira A.C. (2017). Liver Sinusoidal Endothelial Cell: An Update. Semin. Liver Dis..

[18] Fraser R., Dobbs B.R., Rogers G.W. (1995). Lipoproteins and the liver sieve: The role of the fenestrated sinusoidal endothelium in lipoprotein metabolism, atherosclerosis, and cirrhosis. Hepatology.

[19] Hilmer S.N., Cogger V.C., Fraser R., McLean A.J., Sullivan D., Le Couteur D.G. (2005). Age-related changes in the hepatic sinusoidal endothelium impede lipoprotein transfer in the rat. Hepatology.

[20] Herrnberger L., Hennig R., Kremer W., Hellerbrand C., Goepferich A., Kalbitzer H.R., Tamm E.R. (2014). Formation of fenestrae in murine liver sinusoids depends on plasmalemma vesicle-associated protein and is required for lipoprotein passage. PLoS ONE.

[21] Van Berkel T.J., De Rijke Y.B., Kruijt J.K. (1991). Different fate in vivo of oxidatively modified low density lipoprotein and acetylated low density lipoprotein in rats. Recognition by various scavenger receptors on Kupffer and endothelial liver cells. J. Biol. Chem..

[22] Li R., Oteiza A., Sørensen K.K., McCourt P., Olsen R., Smedsrød B., Svistounov D. (2011). Role of liver sinusoidal endothelial cells and stabilins in elimination of oxidized low-density lipoproteins. Am. J. Physiol. Gastrointest. Liver Physiol..

[23] Lopes-Virella M.F., Virella G., Orchard T.J., Koskinen S., Evans R.W., Becker D.J., Forrest K.Y. (1999). Antibodies to oxidized LDL and LDL-containing immune complexes as risk factors for coronary artery disease in diabetes mellitus. Clin. Immunol..

[24] Steinberg D. (1997). Low density lipoprotein oxidation and its pathobiological significance. J. Biol. Chem..

[25] Cogger V.C., Mohamad M., Solon-Biet S.M., Senior A.M., Warren A., O’Reilly J.N., Tung B.T., Svistounov D., McMahon A.C., Fraser R. (2016). Dietary macronutrients and the aging liver sinusoidal endothelial cell. Am. J. Physiol. Heart Circ. Physiol..

[26] Miyao M., Kotani H., Ishida T., Kawai C., Manabe S., Abiru H., Tamaki K. (2015). Pivotal role of liver sinusoidal endothelial cells in NAFLD/NASH progression. Lab. Investig..

[27] Kus E., Kaczara P., Czyzynska-Cichon I., Szafranska K., Zapotoczny B., Kij A., Sowinska A., Kotlinowski J., Mateuszuk L., Czarnowska E. (2019). LSEC fenestrae are preserved despite pro-inflammatory phenotype of liver sinusoidal endothelial cells in mice on high fat diet. Front. Physiol..

[28] Zhang Q., Liu J., Liu J., Huang W., Tian L., Quan J., Wang Y., Niu R. (2014). oxLDL induces injury and defenestration of human liver sinusoidal endothelial cells via LOX1. J. Mol. Endocrinol..

[29] O’Reilly J.N., Cogger V.C., Fraser R., Le Couteur D.G. (2010). The effect of feeding and fasting on fenestrations in the liver sinusoidal endothelial cell. Pathology.

[30] Wieland A., Frank D.N., Harnke B., Bambha K. (2015). Systematic review: Microbial dysbiosis and nonalcoholic fatty liver disease. Aliment. Pharmacol. Ther..

[31] Dobbs B.R., Rogers G.W., Xing H.Y., Fraser R. (1994). Endotoxin-induced defenestration of the hepatic sinusoidal endothelium: A factor in the pathogenesis of cirrhosis?. Liver.

[32] Marra F., Svegliati-Baroni G. (2018). Lipotoxicity and the gut-liver axis in NASH pathogenesis. J. Hepatol..

[33] Francque S., Verrijken A., Mertens I., Hubens G., Van Marck E., Pelckmans P., Van Gaal L., Michielsen P. (2010). Noncirrhotic human nonalcoholic fatty liver disease induces portal hypertension in relation to the histological degree of steatosis. Eur. J. Gastroenterol. Hepatol..

[34] Francque S., Laleman W., Verbeke L., Van Steenkiste C., Casteleyn C., Kwanten W., Van Dyck C., D’Hondt M., Ramon A., Vermeulen W. (2012). Increased intrahepatic resistance in severe steatosis: Endothelial dysfunction, vasoconstrictor overproduction and altered microvascular architecture. Lab. Investig..

[35] Mendes F.D., Suzuki A., Sanderson S.O., Lindor K.D., Angulo P. (2012). Prevalence and indicators of portal hypertension in patients with nonalcoholic fatty liver disease. Clin. Gastroenterol. Hepatol..

[36] Baffy G. (2018). Origins of portal hypertension in nonalcoholic fatty liver disease. Dig. Dis. Sci..

[37] Ijaz S., Yang W., Winslet M.C., Seifalian A.M. (2003). Impairment of hepatic microcirculation in fatty liver. Microcirculation.

[38] Flammer A.J., Anderson T., Celermajer D.S., Creager M.A., Deanfield J., Ganz P., Hamburg N.M., Lüscher T.F., Shechter M., Taddei S. (2012). The assessment of endothelial function: From research into clinical practice. Circulation.

[39] Tateya S., Rizzo N.O., Handa P., Cheng A.M., Morgan-Stevenson V., Daum G., Clowes A.W., Morton G.J., Schwartz M.W., Kim F. (2011). Endothelial NO/cGMP/VASP signaling attenuates Kupffer cell activation and hepatic insulin resistance induced by high-fat feeding. Diabetes.

[40] Pasarín M., La Mura V., Gracia-Sancho J., García-Calderó H., Rodríguez-Vilarrupla A., García-Pagán J.C., Bosch J., Abraldes J.G. (2012). Sinusoidal endothelial dysfunction precedes inflammation and fibrosis in a model of NAFLD. PLoS ONE.

[41] Gonzalez-Paredes F.J., Hernández Mesa G., Morales Arraez D., Marcelino Reyes R., Abrante B., Diaz-Flores F., Salido E., Quintero E., Hernández-Guerra M. (2016). Contribution of cyclooxygenase end products and oxidative stress to intrahepatic endothelial dysfunction in early non-alcoholic fatty liver disease. PLoS ONE.

[42] García-Lezana T., Raurell I., Bravo M., Torres-Arauz M., Salcedo M.T., Santiago A., Schoenenberger A., Manichanh C., Genescà J., Martell M. (2018). Restoration of a healthy intestinal microbiota normalizes portal hypertension in a rat model of nonalcoholic steatohepatitis. Hepatology.

[43] Montagnani M., Chen H., Barr V.A., Quon M.J. (2001). Insulin-stimulated activation of eNOS is independent of Ca2+ but requires phosphorylation by Akt at Ser (1179). J. Biol. Chem..

[44] Matsumoto M., Zhang J., Zhang X., Liu J., Jiang J.X., Yamaguchi K., Taruno A., Katsuyama M., Iwata K., Ibi M. (2018). The NOX1 isoform of NADPH oxidase is involved in dysfunction of liver sinusoids in nonalcoholic fatty liver disease. Free Radic. Biol. Med..

[45] Schild L., Dombrowski F., Lendeckel U., Schulz C., Gardemann A., Keilhoff G. (2008). Impairment of endothelial nitric oxide synthase causes abnormal fat and glycogen deposition in liver. Biochim. Biophys. Acta.

[46] Rui L. (2014). Energy metabolism in the liver. Compr. Physiol..

[47] Doulias P.T., Tenopoulou M., Greene J.L., Raju K., Ischiropoulos H. (2013). Nitric oxide regulates mitochondrial fatty acid metabolism through reversible protein S-nitrosylation. Sci. Signal..

[48] Knolle P.A., Schmitt E., Jin S., Germann T., Duchmann R., Hegenbarth S., Gerken G., Lohse A.W. (1999). Induction of cytokine production in naive CD4(+) T cells by antigen-presenting murine liver sinusoidal endothelial cells but failure to induce differentiation toward Th1 cells. Gastroenterology.

[49] Limmer A., Ohl J., Kurts C., Ljunggren H.G., Reiss Y., Groettrup M., Momburg F., Arnold B., Knolle P.A. (2000). Efficient presentation of exogenous antigen by liver endothelial cells to CD8+ T cells results in antigen-specific T-cell tolerance. Nat. Med..

[50] Yao Z., Mates J.M., Cheplowitz A.M., Hammer L.P., Maiseyeu A., Phillips G.S., Wewers M.D., Rajaram M.V., Robinson J.M., Anderson C.L. (2016). Blood-borne lipopolysaccharide is rapidly eliminated by liver sinusoidal endothelial cells via high-density lipoprotein. J. Immunol..

[51] Uhrig A., Banafsche R., Kremer M., Hegenbarth S., Hamann A., Neurath M., Gerken G., Limmer A., Knolle P.A. (2005). Development and functional consequences of LPS tolerance in sinusoidal endothelial cells of the liver. J. Leukoc. Biol..

[52] Martin-Armas M., Simon-Santamaria J., Pettersen I., Moens U., Smedsrød B., Sveinbjørnsson B. (2006). Toll-like receptor 9 (TLR9) is present in murine liver sinusoidal endothelial cells (LSECs) and mediates the effect of CpG-oligonucleotides. J. Hepatol..

[53] Wu J., Meng Z., Jiang M., Zhang E., Trippler M., Broering R., Bucchi A., Krux F., Dittmer U., Yang D. (2010). Toll-like receptor-induced innate immune responses in non-parenchymal liver cells are cell type-specific. Immunology.

[54] Marra F., Tacke F. (2014). Roles for chemokines in liver disease. Gastroenterology.

[55] Miyachi Y., Tsuchiya K., Komiya C., Shiba K., Shimazu N., Yamaguchi S., Deushi M., Osaka M., Inoue K., Sato Y. (2017). Roles for cell-cell adhesion and contact in obesity-induced hepatic myeloid cell accumulation and glucose intolerance. Cell Rep..

[56] Shetty S., Lalor P.F., Adams D.H. (2018). Liver sinusoidal endothelial cells—gatekeepers of hepatic immunity. Nat. Rev. Gastroenterol. Hepatol..

[57] Wong J., Johnston B., Lee S.S., Bullard D.C., Smith C.W., Beaudet A.L., Kubes P. (1997). A minimal role for selectins in the recruitment of leukocytes into the inflamed liver microvasculature. J. Clin. Investig..

[58] Adams D.H., Hubscher S.G., Fisher N.C., Williams A., Robinson M. (1996). Expression of e-selectin and e-selectin ligands in human liver inflammation. Hepatology.

[59] Lalor P.F., Sun P.J., Weston C.J., Martin-Santos A., Wakelam M.J., Adams D.H. (2007). Activation of vascular adhesion protein-1 on liver endothelium results in an NF-kappaB-dependent increase in lymphocyte adhesion. Hepatology.

[60] Aspinall A.I., Curbishley S.M., Lalor P.F., Weston C.J., Blahova M., Liaskou E., Adams R.M., Holt A.P., Adams D.H. (2010). CX(3)CR1 and vascular adhesion protein-1-dependent recruitment of CD16(+) monocytes across human liver sinusoidal endothelium. Hepatology.

[61] Patten D.A., Wilson G.K., Bailey D., Shaw R.K., Jalkanen S., Salmi M., Rot A., Weston C.J., Adams D.H., Shetty S. (2017). Human liver sinusoidal endothelial cells promote intracellular crawling of lymphocytes during recruitment: A new step in migration. Hepatology.

[62] Weston C.J., Shepherd E.L., Claridge L.C., Rantakari P., Curbishley S.M., Tomlinson J.W., Hubscher S.G., Reynolds G.M., Aalto K., Anstee Q.M. (2015). Vascular adhesion protein-1 promotes liver inflammation and drives hepatic fibrosis. J. Clin. Investig..

[63] Lefere S., Van de Velde F., Devisscher L., Bekaert M., Raevens S., Verhelst X., Van Nieuwenhove Y., Praet M., Hoorens A., Van Steenkiste C. (2017). Serum vascular cell adhesion molecule-1 predicts significant liver fibrosis in non-alcoholic fatty liver disease. Int. J. Obes..

[64] Rai R.P., Liu Y., Iyer S.S., Liu S., Gupta B., Desai C., Kumar P., Smith T., Singhi A.D., Nusrat A. (2020). Blocking integrin α(4)β(7)-mediated CD4 T cell recruitment to the intestine and liver protects mice from western diet-induced non-alcoholic steatohepatitis. J. Hepatol..

[65] Farhood A., McGuire G.M., Manning A.M., Miyasaka M., Smith C.W., Jaeschke H. (1995). Intercellular adhesion molecule 1 (ICAM-1) expression and its role in neutrophil-induced ischemia-reperfusion injury in rat liver. J. Leukoc. Biol..

[66] Douds A.C., Lim A.G., Jazrawi R.P., Finlayson C., Maxwell J.D. (1997). Serum intercellular adhesion molecule-1 in alcoholic liver disease and its relationship with histological disease severity. J. Hepatol..

[67] Essani N.A., Fisher M.A., Farhood A., Manning A.M., Smith C.W., Jaeschke H. (1995). Cytokine-induced upregulation of hepatic intercellular adhesion molecule-1 messenger RNA expression and its role in the pathophysiology of murine endotoxin shock and acute liver failure. Hepatology.

[68] Jaeschke H., Farhood A., Bautista A.P., Spolarics Z., Spitzer J.J., Smith C.W. (1993). Functional inactivation of neutrophils with a Mac-1 (CD11b/CD18) monoclonal antibody protects against ischemia-reperfusion injury in rat liver. Hepatology.

[69] Rosenberg P., Sjöström M., Söderberg C., Kinnman N., Stål P., Hultcrantz R. (2011). Attenuated liver fibrosis after bile duct ligation and defective hepatic stellate cell activation in neural cell adhesion molecule knockout mice. Liver Int..

[70] Bonder C.S., Norman M.U., Swain M.G., Zbytnuik L.D., Yamanouchi J., Santamaria P., Ajuebor M., Salmi M., Jalkanen S., Kubes P. (2005). Rules of recruitment for Th1 and Th2 lymphocytes in inflamed liver: A role for alpha-4 integrin and vascular adhesion protein-1. Immunity.

[71] Kurkijärvi R., Adams D.H., Leino R., Möttönen T., Jalkanen S., Salmi M. (1998). Circulating form of human vascular adhesion protein-1 (VAP-1): Increased serum levels in inflammatory liver diseases. J. Immunol..

[72] Pober J.S., Sessa W.C. (2007). Evolving functions of endothelial cells in inflammation. Nat. Rev. Immunol..

[73] Carmeliet P. (2003). Angiogenesis in health and disease. Nat. Med..

[74] Ding B.S., Nolan D.J., Butler J.M., James D., Babazadeh A.O., Rosenwaks Z., Mittal V., Kobayashi H., Shido K., Lyden D. (2010). Inductive angiocrine signals from sinusoidal endothelium are required for liver regeneration. Nature.

[75] Sato T., El-Assal O.N., Ono T., Yamanoi A., Dhar D.K., Nagasue N. (2001). Sinusoidal endothelial cell proliferation and expression of angiopoietin/Tie family in regenerating rat liver. J. Hepatol..

[76] Yang L., Kwon J., Popov Y., Gajdos G.B., Ordog T., Brekken R.A., Mukhopadhyay D., Schuppan D., Bi Y., Simonetto D. (2014). Vascular endothelial growth factor promotes fibrosis resolution and repair in mice. Gastroenterology.

[77] Öztürk Akcora B., Storm G., Prakash J., Bansal R. (2017). Tyrosine kinase inhibitor BIBF1120 ameliorates inflammation, angiogenesis and fibrosis in CCl(4)-induced liver fibrogenesis mouse model. Sci. Rep..

[78] Tugues S., Fernandez-Varo G., Muñoz-Luque J., Ros J., Arroyo V., Rodés J., Friedman S.L., Carmeliet P., Jiménez W., Morales-Ruiz M. (2007). Antiangiogenic treatment with sunitinib ameliorates inflammatory infiltrate, fibrosis, and portal pressure in cirrhotic rats. Hepatology.

[79] Coulon S., Legry V., Heindryckx F., Van Steenkiste C., Casteleyn C., Olievier K., Libbrecht L., Carmeliet P., Jonckx B., Stassen J.M. (2013). Role of vascular endothelial growth factor in the pathophysiology of nonalcoholic steatohepatitis in two rodent models. Hepatology.

[80] Coulon S., Francque S., Colle I., Verrijken A., Blomme B., Heindryckx F., De Munter S., Prawitt J., Caron S., Staels B. (2012). Evaluation of inflammatory and angiogenic factors in patients with non-alcoholic fatty liver disease. Cytokine.

[81] Papageorgiou M.V., Hadziyannis E., Tiniakos D., Georgiou A., Margariti A., Kostas A., Papatheodoridis G.V. (2017). Serum levels of vascular endothelial growth factor in non-alcoholic fatty liver disease. Ann. Gastroenterol..

[82] Lefere S., Van de Velde F., Hoorens A., Raevens S., Van Campenhout S., Vandierendonck A., Neyt S., Vandeghinste B., Vanhove C., Debbaut C. (2019). Angiopoietin-2 promotes pathological angiogenesis and Is a therapeutic target in murine nonalcoholic fatty liver disease. Hepatology.

[83] Tsuchida T., Friedman S.L. (2017). Mechanisms of hepatic stellate cell activation. Nat. Rev. Gastroenterol. Hepatol..

[84] Kostallari E., Shah V.H. (2016). Angiocrine signaling in the hepatic sinusoids in health and disease. Am. J. Physiol. Gastrointest. Liver Physiol..

[85] Deleve L.D., Wang X., Guo Y. (2008). Sinusoidal endothelial cells prevent rat stellate cell activation and promote reversion to quiescence. Hepatology.

[86] Hilscher M.B., Sehrawat T., Arab J.P., Zeng Z., Gao J., Liu M., Kostallari E., Gao Y., Simonetto D.A., Yaqoob U. (2019). Mechanical stretch increases expression of CXCL1 in liver sinusoidal endothelial cells to recruit neutrophils, generate sinusoidal microthombi, and promote portal hypertension. Gastroenterology.

[87] Poisson J., Lemoinne S., Boulanger C., Durand F., Moreau R., Valla D., Rautou P.E. (2017). Liver sinusoidal endothelial cells: Physiology and role in liver diseases. J. Hepatol..

[88] DeLeve L.D., Wang X., Kanel G.C., Atkinson R.D., McCuskey R.S. (2008). Prevention of hepatic fibrosis in a murine model of metabolic syndrome with nonalcoholic steatohepatitis. Am. J. Pathol..

[89] Wells R.G. (2008). Cellular sources of extracellular matrix in hepatic fibrosis. Clin. Liver Dis..

[90] Wang R., Ding Q., Yaqoob U., de Assuncao T.M., Verma V.K., Hirsova P., Cao S., Mukhopadhyay D., Huebert R.C., Shah V.H. (2015). Exosome adherence and internalization by hepatic stellate cells triggers sphingosine 1-phosphate-dependent migration. J. Biol. Chem..

[91] Xie G., Choi S.S., Syn W.K., Michelotti G.A., Swiderska M., Karaca G., Chan I.S., Chen Y., Diehl A.M. (2013). Hedgehog signalling regulates liver sinusoidal endothelial cell capillarisation. Gut.

[92] Hirsova P., Ibrahim S.H., Bronk S.F., Yagita H., Gores G.J. (2013). Vismodegib suppresses TRAIL-mediated liver injury in a mouse model of nonalcoholic steatohepatitis. PLoS ONE.

[93] Ding B.S., Cao Z., Lis R., Nolan D.J., Guo P., Simons M., Penfold M.E., Shido K., Rabbany S.Y., Rafii S. (2014). Divergent angiocrine signals from vascular niche balance liver regeneration and fibrosis. Nature.

[94] van Niel G., D’Angelo G., Raposo G. (2018). Shedding light on the cell biology of extracellular vesicles. Nat. Rev. Mol. Cell Biol..

[95] Yáñez-Mó M., Siljander P.R., Andreu Z., Zavec A.B., Borràs F.E., Buzas E.I., Buzas K., Casal E., Cappello F., Carvalho J. (2015). Biological properties of extracellular vesicles and their physiological functions. J. Extracell. Vesicles.

[96] Ibrahim S.H., Hirsova P., Gores G.J. (2018). Non-alcoholic steatohepatitis pathogenesis: Sublethal hepatocyte injury as a driver of liver inflammation. Gut.

[97] Hirsova P., Ibrahim S.H., Verma V.K., Morton L.A., Shah V.H., LaRusso N.F., Gores G.J., Malhi H. (2016). Extracellular vesicles in liver pathobiology: Small particles with big impact. Hepatology.

[98] Verweij F.J., Revenu C., Arras G., Dingli F., Loew D., Pegtel D.M., Follain G., Allio G., Goetz J.G., Zimmermann P. (2019). Live tracking of inter-organ communication by endogenous exosomes in vivo. Dev. Cell.

[99] Witek R.P., Yang L., Liu R., Jung Y., Omenetti A., Syn W.K., Choi S.S., Cheong Y., Fearing C.M., Agboola K.M. (2009). Liver cell-derived microparticles activate hedgehog signaling and alter gene expression in hepatic endothelial cells. Gastroenterology.

[100] Lemoinne S., Cadoret A., Rautou P.E., El Mourabit H., Ratziu V., Corpechot C., Rey C., Bosselut N., Barbu V., Wendum D. (2015). Portal myofibroblasts promote vascular remodeling underlying cirrhosis formation through the release of microparticles. Hepatology.

[101] Povero D., Eguchi A., Niesman I.R., Andronikou N., de Mollerat du Jeu X., Mulya A., Berk M., Lazic M., Thapaliya S., Parola M. (2013). Lipid-induced toxicity stimulates hepatocytes to release angiogenic microparticles that require Vanin-1 for uptake by endothelial cells. Sci. Signal..

[102] Mittal S., Sada Y.H., El-Serag H.B., Kanwal F., Duan Z., Temple S., May S.B., Kramer J.R., Richardson P.A., Davila J.A. (2015). Temporal trends of nonalcoholic fatty liver disease-related hepatocellular carcinoma in the veteran affairs population. Clin. Gastroenterol. Hepatol..

[103] Younes R., Bugianesi E. (2018). Should we undertake surveillance for HCC in patients with NAFLD?. J. Hepatol..

[104] Sanyal A., Poklepovic A., Moyneur E., Barghout V. (2010). Population-based risk factors and resource utilization for HCC: US perspective. Curr. Med. Res. Opin..

[105] Mittal S., El-Serag H.B., Sada Y.H., Kanwal F., Duan Z., Temple S., May S.B., Kramer J.R., Richardson P.A., Davila J.A. (2016). Hepatocellular carcinoma in the absence of cirrhosis in United States veterans is associated with nonalcoholic fatty liver disease. Clin. Gastroenterol. Hepatol..

[106] Medina J., Arroyo A.G., Sanchez-Madrid F., Moreno-Otero R. (2004). Angiogenesis in chronic inflammatory liver disease. Hepatology.

[107] Zhuang P.Y., Wang J.D., Tang Z.H., Zhou X.P., Quan Z.W., Liu Y.B., Shen J. (2015). Higher proliferation of peritumoral endothelial cells to IL-6/sIL-6R than tumoral endothelial cells in hepatocellular carcinoma. BMC Cancer.

[108] Geraud C., Mogler C., Runge A., Evdokimov K., Lu S., Schledzewski K., Arnold B., Hammerling G., Koch P.S., Breuhahn K. (2013). Endothelial transdifferentiation in hepatocellular carcinoma: Loss of Stabilin-2 expression in peri-tumourous liver correlates with increased survival. Liver Int..

[109] Boord J.B., Maeda K., Makowski L., Babaev V.R., Fazio S., Linton M.F., Hotamisligil G.S. (2004). Combined adipocyte-macrophage fatty acid-binding protein deficiency improves metabolism, atherosclerosis, and survival in apolipoprotein E-deficient mice. Circulation.

[110] Hotamisligil G.S., Johnson R.S., Distel R.J., Ellis R., Papaioannou V.E., Spiegelman B.M. (1996). Uncoupling of obesity from insulin resistance through a targeted mutation in aP2, the adipocyte fatty acid binding protein. Science.

[111] Maeda K., Cao H., Kono K., Gorgun C.Z., Furuhashi M., Uysal K.T., Cao Q., Atsumi G., Malone H., Krishnan B. (2005). Adipocyte/macrophage fatty acid binding proteins control integrated metabolic responses in obesity and diabetes. Cell Metab..

[112] Hancke K., Grubeck D., Hauser N., Kreienberg R., Weiss J.M. (2010). Adipocyte fatty acid-binding protein as a novel prognostic factor in obese breast cancer patients. Breast Cancer Res. Treat..

[113] Nieman K.M., Kenny H.A., Penicka C.V., Ladanyi A., Buell-Gutbrod R., Zillhardt M.R., Romero I.L., Carey M.S., Mills G.B., Hotamisligil G.S. (2011). Adipocytes promote ovarian cancer metastasis and provide energy for rapid tumor growth. Nat. Med..

[114] Milner K.L., van der Poorten D., Xu A., Bugianesi E., Kench J.G., Lam K.S., Chisholm D.J., George J. (2009). Adipocyte fatty acid binding protein levels relate to inflammation and fibrosis in nonalcoholic fatty liver disease. Hepatology.

[115] Laouirem S., Sannier A., Norkowski E., Cauchy F., Doblas S., Rautou P.E., Albuquerque M., Garteiser P., Sognigbe L., Raffenne J. (2019). Endothelial fatty liver binding protein 4: A new targetable mediator in hepatocellular carcinoma related to metabolic syndrome. Oncogene.

